# 

**Published:** 1876-07

**Authors:** 


### CONTENTS:

### ORIGINAL COMMUNICATIONS.

ARTICLE I.—Mechanical Dentistry—Its present condition. &c. By Dr. L. P. Haskell.......... 97
ARTICLE II.—Dental Therapeutics. By S. H. Henkel, D. D. S.......... 105
ARTICLE III—Treatment of Exposed Pulps. By C. C. Chittenden.......... 110
New York State Dental Society.......... 115
American Academy of Dental Science.......... 115

### SELECTED ARTICLES.

ARTICLE IV.—On Toothache.......... 116

ARTICLE V.—Celluloid.......... 122

ARTICLE VI.—Washington's Teeth.......... 127

ARTICLE VII.—Epulis of Lower Jaw.......... 132

ARTICLE VIII.—On Treatment of Convulsions in Infants.......... 133

### EDITORIAL, ETC.

Reform in Medical Colleges.......... 135
Help for the Deserving.......... 136

### MONTHLY SUMMARY.

A More Effectual Mode of Applying Iodine to the Interior or Certain Cysts.......... 138

Some Health Principles.......... 139

Treatment of Cysts.......... 140

Treatment of Children in Egypt.......... 140

Vegetable Diet in relation to Longevity.......... 141

An Extraordinary Story.......... 141

Treatment of Stammering.......... 141

Nitrite of Amly.......... 142

Carbolic Acid Sprays in Throat Affections.......... 142

Natural Ink.......... 143

How to Cure a Cold in the Head.......... 143

Eye-sight.......... 143

Wounds.......... 144

Nervous Headache and Neuralgia.......... 144

Soft Hands.......... 144

